# Adherence to Antiretroviral Therapy (ART) among People Living With HIV (PLHIV): a cross-sectional survey to measure in Lao PDR

**DOI:** 10.1186/1471-2458-13-617

**Published:** 2013-06-28

**Authors:** Visanou Hansana, Pattara Sanchaisuriya, Jo Durham, Vanphanom Sychareun, Kongmany Chaleunvong, Suwanna Boonyaleepun, Frank Peter Schelp

**Affiliations:** 1 Faculty of Postgraduate studies, University of Health Sciences, Vientiane, Lao PDR; 2 Faculty of Basic Sciences, University of Health Sciences, Vientiane, Lao PDR; 3 Faculty of Public Health, KhoneKaen University, KhoneKaen, Thailand; 4 Faculty of Nursing, KhoneKaen University, KhoneKaen, Thailand; 5 University of Queensland, School of Population Health, Australian Centre of International and Tropical Health, Brisbane, QLD, Australia

**Keywords:** Antiretroviral therapy, Adherence, PLWHIV, Self-report, Lao PDR

## Abstract

**Rationale:**

Since 2001, antiretroviral therapy (ART) for people living with HIV (PLHIV) has been available in the Lao People’s Democratic Republic (PDR). A key factor in the effectiveness of ART is good adherence to the prescribed regimen for both individual well-being and public health. Poor adherence can contribute to the emergence of drug resistant strains of the virus and transmission during risky behaviors. Increased access to ART in low-income country settings has contributed to an interest in treatment adherence in resource–poor contexts. This study aims to investigate the proportion of adherence to ART and identify possible factors related to non-adherence to ART among people living with HIV (PLHIV) in Lao PDR.

**Methods:**

A cross-sectional study was conducted with adults living with HIV receiving free ART at Setthathirath hospital in the capital Vientiane and Savannakhet provincial hospitals from June to November 2011. Three hundred and forty six PLHIV were interviewed using an anonymous questionnaire. The estimation of the adherence rate was based on the information provided by the PLHIV about the intake of medicine during the previous three days. The statistical software Epidata 3.1 and Stata 10.1 were used for data analysis. Frequencies and distribution of each variable were calculated by conventional statistical methods. The chi square test, Mann–Whitney test and logistic regression were used for bivariate analyses. Multiple logistic regression analysis was conducted to determine the predictors of non-adherence to ART. A p-value < 0.05 was considered to indicate statistical significance.

**Results:**

Of a total of 346 patients, 60% reported more than 95% adherence to ART. Reasons for not taking medicine as required were being busy (97.0%), and being forgetful (62.2%). In the multivariate analysis, educational level at secondary school (OR=3.7, 95% CI:1.3-10.1, p=0.012); illicit drug use (OR=16.1, 95% CI:1.9-128.3, p=0.011); dislike exercise (OR=0.6, 95% CI:0.4-0.9, p=0.028), and forgetting to take ARV medicine during the last month (OR=2.3, 95% CI:1.4-3.7, p=0.001) were independently associated with non-adherence.

**Conclusions:**

Non-adherence to ART was associated with individual factors and exposure to ART. Priority measures to increase adherence to ART should aim to intensify counseling and comprehensive interventions, such as guidance for PLHIV on medication self-management skills, tailoring the regimen to the PLHIV life style, and improving adherence monitoring and health care services.

## Background

The introduction of antiretroviral therapy (ART) and multidrug regimens, or highly active antiretroviral therapy (HAART) has substantially improved the survival of persons infected with HIV. These drug regimens however are complex. This, alongside issues of toxicity, side-effects, disruptions to patient’s daily life and difficulties in returning for scheduled follow-up consultations, often makes maintaining adherence over the long term challenging. Yet the individual and public health benefits of ART are adherence dependent. Inadequate adherence results in antiretroviral agents not being maintained at sufficient concentrations to suppress HIV replication in infected cells to lower the plasma viral load. In addition, suboptimal adherence can accelerate development of drug-resistant HIV and mitigate ART’s role in reducing HIV incidence and transmission. Promoting adherence is especially important as these treatments become increasingly available and affordable for people living with HIV (PLHIV) in developing countries where viral load monitoring is not usually possible [1,2]. Consequently, more attention is being focused on issues related to ART adherence.

The importance of ART adherence makes accurate compliance assessment essential for effective and efficient therapy and evaluation of treatment regimens [3,4]. There are a number of key issues in the study of ART adherence including having an accurate measure of adherence [5-7]. Measurement of adherence is usually based on Paterson’s pioneer study found that up to 95% adherence is necessary for effective HIV viral suppression [3]. This measurement is usually obtained by using either a continuous or categorical variable constructed from patient self-reported adherence that distinguishes “optimal” from “sub-optimal” adherence based on the 95% threshold [8-10]. A limitation of this method is it only reflects short-term or average adherence and may be an over-estimation. Nevertheless, studies have demonstrated a significant relationship between self-report data and viral load [8-10].

Besides accurate measurement, other factors of importance in the study of ART adherence include sassessment of the impact of adherence on viral load and clinical outcome, identification of the factors that affect adherence and effective interventions. Understanding the factors that affect non-adherence could provide valuable information about patients most at risk. Studies suggest that factors associated with adherence can be grouped under four main categories. One relates to patient factors, for example demographic characteristics [11]. Another relates to psychological parameter knowledge, personal skills. A third category relates to treatment regimen-related factors such as years on treatment, pill burden, side effects; provider-related factors including the patient-provider relationship [12]. The fourth category relates to environmental and social factors such as supervision of treatment, HIV-related stigma and social support [6,13]. While some factors determining non-adherence to ART may be similar across countries, others may be highly contextual, and culture or country specific [7,8].

Most ART adherence studies have been undertaken in high-income countries [5-7]. Few studies have assessed adherence rates or predictors in low-or lower-middle income countries. This paper presents the findings of a study which investigated adherence to ART and possible factors related to non-adherence among PLHIV receiving ART in two public health facilities in the Lao People’s Democratic Republic (PDR), a lower-middle income country in South East Asia. As far as the authors are aware, this is the first such study in Lao PDR. The study fills a critical gap in understanding ART adherence in Lao PDR and contributes to the growing adherence research in low-income countries. Such studies are vital to ensure decisions about adherence interventions are informed by empirical support.

The prevalence of HIV among adults in Lao PDR is low (0.3%) [14], nevertheless, the number of PLHIV is increasing. It is estimated that approximately 10,000 people are living with HIV in Lao PDR with the first case identified in 1990 [1]. The first specific HIV/AIDS project in Lao PDR was started by Medecins San Frontiers (MSF) in Savannakhet province, in 2001. This program offered free service, prophylaxis and treatment for opportunistic infections (OIs), and ART [15]. Presently, there are five treatment centers and two satellite sites providing ART in the country. Two of these centers are in the capital Vientiane; two are in the southern provinces of Savannakhet and Champasack. The fifth center is in Luangphabang province in the north. There are also two satellite sites in the north (Borkeo and Luangnamtha provinces), also in the north and funded by the Global Fund to fight AIDS, TB and Malaria (GFATM). While the program has extended since the first case was identified, accesses to treatment for PLHIV by the end of 2011, total of 1,988 adults and children PLHIV who were in need have received ART. This is equivalent to 52.3% of estimated PLHIV eligible for ARV have received ART, a small increase compare to 50.8% in 2010 [16]. For 2010 and 2011, despite continuous increase in the absolute number of PLHIV who received ART, the percentage rate dropped due to change in calculation for estimate eligible PLHIV for ART [17].

## Methods

### Study setting

This study was conducted at the Setthathirath, tertiary level hospital located in the Vientiane capital and Savannakhet provincial hospital. These two geographical areas have the greatest HIV burden and are currently the two main sites providing free ART and related health care services. These facilities were selected as they were the most established clinics providing ART in Lao PDR, had a pool of HIV-infected patients on ART and were willing to participate in the study. At the time of the study in Savannakhet provincial hospital 1,447 HIV patients were registered of which 1,106 were receiving ART medicines [18]. In Setthathirath hospital 821 HIV patients were registered of which 595 were on ART [19].

### Study design and participants

This cross-sectional study was performed from January to March 2011 in Savannakhet and Sethathirath hospitals. Participants in the study were PLHIV receiving free ART provided by the HIV/AIDS care unit. To be included participants had to be 18 years or over and willing to participate in the study. The clinical condition of the patient had to be suitable for ART based on the CD_4_ count and to be in the WHO clinical stage for at least three months. The PLHIV were responsible for how and when to take the medicine.

### Sampling

PLHIV receiving ART were selected consecutively from both study sites by using probability proportional to size, based on the number of PLHIV who were on ART. The participants were selected using simple random sampling. Participants were drawn either from the PLHIV on ART list at the care unit or during a visit to the HIV/AIDS care unit for counseling and/or ART medication. The initial sample size was estimated to be 305 with a power of 0.84. However, we were able to enroll 346 participants, 130 from Sethathirath hospital and 216 from Savannakhet provincial hospital, 150 were recruited from the PLHIV on ART list and 196 came for regular visit. Four respondents declined to be interviewed as part of the study.

### Measurement

Adherence measurement in this study was based on patient recall of their compliance of the prescribed doses in the three days prior to the interview. Adherence was calculated based on the number of pills reported to have been actually taken divided by the number of prescribed pills over in the past three days. Patients who reported an intake of ≥ 95% of the prescribed medication were considered adherent, those with a reported intake of < 95% were classified as non-adherent.

### Data collection

A face-to-face interview with PLHIV by two trained interviewers at the HIV/AIDS center was conducted using a structured, self-report questionnaire developed by the Adult AIDS Clinical Trials Group (AACTG) [20] and slightly modified after a pre-testing at Mahosot hospital, Vientiane Capital (*N*=30). The AACTG was developed based on previous adherence to medical regimen research and has been extensively used to measure ART adherence. The questionnaire asked patients to recall their medication intake in terms of their prescribed doses for the three days prior to the interview. It also collected demographic data and information about health behaviors, seriousness of the disease, side effects and adverse drug reactions. In addition, it asked about health service-related characteristics of the center providing ART. Prior to administration, the questionnaire was translated into Lao and back translated by an independent translator. To ensure confidentiality, all respondents were interviewed in a private room at the HIV/AIDS care unit by the trained interviewers.

### Data analysis

The statistical software Epi data 3.1 and Stata 10.1 were used to perform the analysis. The dependent variable was adherence to ART. To assess factors associated with non-adherence to ART, univariate, bivariate and multivariate analyses were conducted. Univariate analysis used conventional statistical methods to calculate frequencies and distribution of each variable. The chi square test and the Mann–Whitney test as well as a logistic regression were used for bivariate analyses. Multivariate analyses were performed to identify factors associated with premarital sexual activities with only those variables with *p*<0.10 from the initial bivariate analyses included in the model and controlling for other confounders such as socio-demographic characteristics of the participants and health risk behaviors (smoking, drinking and doing exercise). A p-value < 0.05 was considered to indicate statistical significance.

### Ethical considerations

The study was undertaken as part of a higher research degree at KhonKaen University, Thailand. The study protocol was approved by the Ethic Committee’s Declaration of the University of Health Sciences, Lao PDR and the Ethics Committee of the KhonKaen University, Thailand. The interviewer explained the purpose of the study and procedures and gained written informed consent before commencing the interview. The participants were also informed that their participation was voluntary and that they could withdraw from the interview/discussion at any time without consequences. The participants were assured that their responses would be treated in confidence and they were assured anonymity through the use of strict coding measures.

## Results

### Socio-demographics

Three hundred and forty six PLHIV were recruited for this study. Of these, 47.4% were female with an age range of 20 to 66 years (Mean ±SD, 36.5±7.6 years). Slightly higher than two fifths of respondents (41.6%) were secondary school graduates, just over half (56.4%) were self employed and received a monthly income ranging from 50,000 to 10,000,000 kip/month (exchange rate 1US$=8,000 LAK). Two hundred and twelve (61.3%) participants were married. Less than half (43.6%) were HIV positive for less than 36 months. The household size ranged from 1 to 15 persons (Mean ±SD: 4.7±2.3 persons). Almost half of the respondents (48.6%) lived with their spouses or partners and only 10 out of the 346 respondents lived alone. Almost all of the respondents (93.6%) had disclosed their HIV status to the people around them (see Table 1).

**Table 1:** Socio-demographic characteristic of respondents

**Socio-demographics**	**Number (n=346)**	**Percentage (%)**
**Sex**		
Male	182	52.6
Female	164	47.4
**Age (year)**	Mean: 36.5±7.6; Min: 20; Max: 66	
≤ 35	166	47.9
36-45	139	40.2
≥46	41	11.9
**Educational attainment**		
Illiterate	32	9.3
Primary school	136	39.3
Secondary school	144	41.6
University	34	9.8
**Working status**		
No job	82	23.7
Self employed	195	56.4
Government staff	18	5.2
Private staff	51	14.7
**Monthly income (Lao kip) (n=264)**	Range: 50,000-10,000,000	
≤ 200,000	66	25.0
200,001-1,000,000	133	50.4
≥1,000,001	65	24.6
**Marital status**		
Single	61	17.6
Married	212	61.3
Separated/Divorced	46	13.3
Widowed	27	7.8
**Duration since HIV diagnosed (months)**	Mean:46.7±30.1; Min: 3; Max: 168	
≤ 36	151	43.6
37-60	90	26.0
>60	105	30.4
**No. of people living in the same house**	Mean:4.7±2.3; Min: 1; Max: 15	
1	10	2.9
2-5	228	65.9
>5	108	31.2
**Person(s) living in the same house**		
Live alone	10	2.9
Parent	110	31.8
Spouse/partners	168	48.6
Children	25	7.2
Relative/friend	33	9.5
**Disclosed HIV status**		
No	22	6.4
Yes	324	93.6

### Health risk behavior characteristics

The health risk behaviors of the respondents included currently smoking (21.7%), drinking alcohol during the last month (43.1%), using illicit drugs (3.5%) and having had multiple sex partners (10.9%). Some gender differences were observed with most male respondents for example, reporting being current smokers while only four percent of women reported being a current smoker (p<0.001). Two thirds (60.9%) of men reported drinking alcohol during the last month, while only twenty percent of women reported consuming alcohol in the month prior to the survey (p<0.001). Two thirds (16.5%) of men reported having multiple sex partners compared to 4.9% of the women (p=0.001). More women than men however reported disliking physical exercise (70% vs 37%, p<0.001). Table 2 shows the reported risk behaviors of respondents.

**Table 2:** Health risk behaviors by sex of respondents

**Behavior**	**Male**	**Female**	**Total**	**P value**
Current smoking	68(37.4)	6(3.7)	74(21.5)	<0.001
Alcohol drinking (last month )	111(60.9)	38(23.2)	149(43.1)	<0.001
Ever used illicit drug	7(3.9)	5(3.1)	12(3.5)	0.686
Ever had multiple sex partners	30(16.5)	8(4.9)	38(10.9)	0.001
Dislike exercise	67(36.8)	114(69.5)	181(52.3)	<0.001

### Medical history and ART treatment

The number of patients who reported being admitted to the hospital after being infected with HIV due to poor health or opportunistic infection was 213(61.6%). The most common reasons for hospital admission were prolonged fever, TB/pneumocystis and chronic diarrhea. Most respondents (91.0%) reported they felt better since starting ART. Only two patients (0.6%) reported that their health had become worse after starting ART. Almost half 154, (44.5%) of the PLHIV were classified as being in the clinical HIV stage IV based on the WHO standard and the CD_4_ count. The mean CD_4_ count was 148.1±175.4 mm^3^ with a range of 2 to 934 with most respondents (88.7%) having CD_4_ cell counts of less than 350 mm^3^ which is the threshold to ART treatment according to the National guidelines [21]. The mean duration of receiving ART was 38.2 ± 25.9 months, with 44.2% having started ART treatment in the 3 to 30 months prior to the survey. The result of this study found that the mean of duration of ART started was 38.2 months with a range from 3 to 99 months.

### Level of non-adherence to ART

Non adherence to the prescribed medication and dosage was reported by 39.1% PLHIV. The major reasons given for non-compliance were being too busy and forgetfulness (97.0% vs 62.2% respectively). Forty three (12.4%) of the respondents reported having missed at least one medical appointment. The reasons for this were given as being too busy (41.9%), lack of money to travel to the health center (32.6%) or because the center was too far from their home (27.9%). One hundred and eighty one (52.3%) patients reported having had experienced side-effects due to their treatment. Of these, the most common symptom was a rash (42%), followed by headache or dizziness (34.3%) and numbness (32.6%) (see Table 3). In order to examine factors associated with adherence, two groups were created. One consisted of those with an adherence score of ≥ 95% (n=206, 59.5%). Participants with adherence scores of ≤ 95% (*n*=140, 40.5%) were allocated to a non-adherence group.

**Table 3:** Medical history and ART of respondents

**Medical history and ART treatment**	**Number (n=346)**	**Percentage (%)**
**Admitted to the hospital related to HIV infection**		
No	133	38.4
Yes	213	61.6
**Reasons for hospital admittance (n=213)**		
Chronic diarrhea	77	36.2
Chronic herpes simplex infection	10	4.7
TB/pneumocystis	91	42.7
Meningitis	24	11.3
Prolong fever	100	46.9
Weight loss	66	30.9
Jaundice	14	6.6
**WHO clinical stage**		
Stage I	22	6.4
Stage II	45	13.0
Stage III	125	36.1
Stage IV	154	44.5
**CD4 at initiation of ART (n=326)**	Mean148.1±175.4; Min: 2; Max: 934	
< 350/mm^3^	289	88.7
≥350/mm^3^	37	11.3
**Duration of ART (months)**	Mean:38.2±25.9; Min: 3; Max: 99	
3-30	153	44.2
31-60	118	34.1
61-99	75	21.7
**Number of ARV medicines received**		
1	67	19.4
≥2	279	80.6
**Health status since initiation of ART**		
Worse	2	0.6
Stable	29	8.4
Better	315	91.0
**Ever forgotten to take ARV medicine in the last month**		
Never	211	60.9
Ever	135	39.1
**Reasons for forgetting to take ARV medicine (n=135)**		
Forgot	84	62.2
Busy	131	97.0
Too many pills/taste of medicine	5	3.7
Long distance to hospital	10	7.4
Run out of medicine	7	5.2
Severe symptoms/difficult to follow-up	6	4.4
Stigma	7	5.2
**Ever had side effects of ARV medicine**		
Never	165	47.7
Ever	181	52.3
**Ever missed an appointment**		
Never	303	87.6
Ever	43	12.4

### Factors associated with ART adherence

The results of the bivariate analysis (Table 4) revealed a statistically significant association with non-adherence in the 36 to 45 age group compared to those who were ≤ 35 years or who were more than 46 years old (p=0.019). Being a graduate of a secondary school was also observed to have a statistically significant association with non-adherence (p=0.008). Patients who had been diagnosed HIV positive for more than 60 months reported statistically significantly better adherence than those who became aware of their HIV infection in the 36 to 45 months (p=0.045) prior to the survey. Use of illegal drug substances was found to be statistically significantly associated with non-adherence (p=0.006), disliking exercise (p=0.025) or having forgotten to take ART medicine during last month (p=0.001).

**Table 4:** Factor associate with antiretroviral therapy (ART) adherence with in the past 3 days (n=346)

**Characteristic**	**Adherence in past 3 days (%)**				*Odds ratio (95% confidence interval)			
	**≥95% (n=206)**		**<95% (n=140)**		**Unadjusted**	***p***	**Adjusted**	***p***
**Age (years)**								
≤ 35	108	(65.1)	58	(34.9)	1			
36-45	72	(51.8)	67	(48.2)	1.7(1.1-2.7)	0.019	-	-
≥46	26	(63.4)	15	(36.6)	1.1(0.5-2.2)	0.834	-	-
**Educational attainment**								
Illiterate	25	(78.1)	7	(21.9)	1			
Primary school	86	(63.2)	50	(36.8)	2.1(0.8-5.1)	0.115	-	-
Secondary school	74	(51.4)	70	(48.6)	3.4(1.4-8.3)	0.008	3.7(1.3-10.1)	0.012
University	21	(61.8)	13	(38.2)	2.2(0.7-6.5)	0.152	-	-
**Duration since HIV diagnosis (month)**								
≤ 36	98	(64.9)	53	(35.1)	1			
37-60	53	(58.9)	37	(41.1)	1.2(0.8-2.2)	0.315	-	-
>60	55	(52.4)	50	(47.6)	1.7(1.1-2.8)	0.045	-	-
**Ever used illicit drugs**								
No	205	(61.4)	129	(46.7)	1			
Yes	1	(8.3)	11	(34.8)	17.5(2.2-137.0)	0.006	16.1(1.9-128.3)	0.011
**Dislike exercise**								
No	88	(53.3)	77	(40.5)	1			
Yes	118	(65.2)	63	(34.8)	0.6(0.4-0.9)	0.025	0.6(0.4-0.9)	0.028
**Forgot to take ARV medicine in the last month**								
Never	141	(66.8)	70	(33.2)	1			
Ever	65	(48.2)	70	(51.8)	2.2(1.4-3.4)	0.001	2.3(1.4-3.7)	0.001
**Miss appointment**								
No	186	(61.4)	117	(38.6)	1		-	-
Yes	20	(41.5)	23	(53.5)	1.8(0.9-3.5)	0.066	-	-

### Multivariate logistic regression model of factors associated with non-adherence to ART

After adjustment for established risk factors for non-adherence (Table 4), factors that remained significantly associated with non-adherence were education attainment at secondary school (OR= 3.7; 95% CI: 1.3-10.1, p = 0.012), illicit drug use (OR = 16.1; 95% CI: 1.9-128.3, p = 0.011), disliking exercise (OR = 0.6; 95% CI:0.4-0.9, p = 0.028), and forgetting to take ART medicine during the last month (OR=2.3; 95% CI:1.4-3.7, p = 0.001).

## Discussion

In this study, we examined factors associated with ART adherence in Lao PDR. Non-adherence usually means skipping one dose of medicine. Based on the self-report questionnaire, the study results indicated that more than half of the PLHIV had good adherence to ART defined as ≥95%. Nevertheless, 40% of respondents reported poor ART adherence. This is a concern given poor adherence severely compromises effectiveness and is linked with the likelihood of the drug resistance. Non-adherence to ART in this study was associated with socio-demographic characteristics, illicit drug use, and history of taking ART. In the final model in our sample, the variables educational level at secondary school, having used illicit drugs, having started ART within the last 31–60 months and forgetfulness played a significant role in predicting adherence to ART.

The finding of 60% ART adherence among PLHIV in our study is similar to that found in a study in Zambia and one in India [22,23], but lower than studies China [24], and Vietnam [25]. It is also lower than reported in studies conducted in developed countries using similar self-report measures [26,27]. Some caution is needed in comparing adherence rates across studies however, as the methods of measuring adherence (self-report vs. pill counts or medication electronic monitoring system (MEMS) and settings such as free ART vs. non-free, rural vs. urban, plasma donor vs. injecting drug users or other risk groups) can affect findings. Measurement of ART adherence is also problematic as patients may overestimate their adherence due to recall bias, the demand characteristics of patient-provider consultation and the desire to avoid criticism [27].

In our study, reasons given for non-adherence related to being busy, forgetfulness and distance to the clinic. Other reasons given included running out of medication, having too many pills to take, the taste of the medication, severe side effects, difficulties in maintaining the medication regiment and self-stigma. Self-stigma may lead to patients being unwilling or fearful of taking medicine when other people are present [24]. A qualitative study in Vietnam identified self-stigma as a pervasive barrier to ART adherence with patients concerned that taking medications in the presence of others could lead to unplanned disclosure of their HIV status [28]. Forgetfulness was identified in our study as a significant variable in predicting adherence to ART. This is similar to a study in rural China [24]. In the present study, we found a statistically significant association between non-adherence and secondary school educational level. Other studies have shown low levels of education as an important factor associated with non-adherence and a general barrier for medical care [29,30]. In contrast, a study in Bangalore, India found education had no effect on adherence [31]. This difference in findings may be due to our participant group in general being relatively well educated and thus more aware about the problems of sub-optimal treatment.

Illicit drug use has been associated with depression and anxiety, either as part of the withdrawal process or because of repeated use. This is particularly relevant in the treatment of HIV infection because depression is one of the strongest predictors of poor adherence and poor treatment outcomes [30]. Our study found that illicit drug use was a contributing factor to non-adherence based on the 95% threshold. Illicit drug use has been reported to reduce the level of adherence in many studies [32-34]. Various studies have documented that widespread illicit drug use among PLHIV makes their treatment complicated and hinders their quality of life [4,35]. It has also been suggested that less than 95% adherence when combined with illicit drug may lead to viral suppression [3].

Being on ART for 31–60 months was significantly associated with non-adherence to ART in this study. This finding is supported by Andreo C. et al. who found that where patients had been on ART medication for more than two years, increased non-adherence was observed [36]. This could be because the longer patients are on treatment they become complacent and find it harder to follow the strict regimen. Research in Vietnam also identified that over time, family members who had initially played a supportive role in helping patients take their medication, assumed the PLHIV was taking their medication as prescribed and followed up with the patient less regularly [28]. This factor may also be a contributing factor in decreased adherence over time.

Some studies have identified side effects of ART medications as significant barriers to good adherence [37]. In this study, although half of the patients (52.3%) reported signs and symptoms of adverse reactions to their treatment, further analysis did not identify these factors as being significantly related to non-adherence. However, this might be due to the limitations of the methods used as both non-adherence and the adherence group reported having trouble with side effects. Nevertheless, it would seem to be a priority that ART programs should where possible, increase the availability of regimens with fewer adverse reactions.

In the present study, no difference in adherence levels based on the duration of taking ART and WHO clinical stages was observed. In addition, the CD_4_ count at the start of treatment did not significantly correlate with non-adherence to ART. A study in India demonstrated an association between self-reported adherence and improvement in CD_4_ counts [31]. According to Sarna et al., [38] lower improvement in CD_4_ counts during the early stages of taking ART medication may be due to a delay between virologic and immunologic failure.

The present study suggests that to increase ART compliance a number of strategies are required. There has been limited research however on the effectiveness of strategies to enhance adherence in low-income settings. Directly observed therapy (DOT) as used in tuberculosis treatment, has been used with ART with some effect but is resource intensive [39]. In Vietnam, perceptions of stigma prevented home-based DOT being viewed as acceptable [28]. Other interventions include limiting the size, number, side effects and frequency of pills taken per day. Such regimens are likely to be more attractive to patients trying to conceal their HIV status for fear of stigma [28].

Mobile phones can provide an inexpensive and convenient means of communication to support treatment compliance. Randomized controlled trials have found mobile phones to be an effective adherence support tool in in low-resource settings [40]. A trial in Kenya for example found a weekly mobile phone text asking in the local language “how are you?” improved viral suppression outcomes when compared with care [41]. In focus group interviews patients reported that the weekly messages made them feel that someone cared. Another trial found that short weekly one-way text messaging improved medication adherence whereas daily messaging did not [42]. A study in Vietnam however, found that while there was overall acceptance of the use of mobile phones, stigma and fear of HIV disclosure were a barrier to use [25]. In Lao PDR, mobile phones are sometimes used to contact the patients when they miss appointments but texting or other mobile phone functions are not currently used to encourage compliance. Given in the present study stigma was a barrier to optimal ART compliance and some HIV patients were also illegal drug-users, further research into the appropriateness of mobile phone use as a form of adherence support in Lao PDR is needed. Issues of confidentiality would also need to be addressed. Research would also be needed to identify the preferred types of mobile phone functions for adherence maintenance. Provider attention to developing a good relationship with the patient, taking time to address concerns about the medications and side-effects can improve adherence. Counseling, education and peer support are other interventions which have been identified as being effective [25]. In Vietnam a randomized controlled trial found peer support improved quality of life and adherence after 12 months among ART patients presenting at clinical stages 3 and 4 at baseline, but had no impact on quality of life among ART patients enrolled at clinical stages 1 and 2 [28].

### Limitations of study

The findings of this study must be interpreted in the light of its limitations. The study was conducted at only two sites in the country and the findings may not be generalizable to other clinical settings. There is no gold standard for measuring adherence and our measurement of adherence is based on PLHIV self-reports of missed doses which may be subject to social desirability and recall biases. The literature for example, suggests that PLHIV tend to overestimate adherence [43]. However, many other studies document that well collected self-reported data clearly correlate with virologic change and is more practical in most settings [44]. A meta-analysis of several studies demonstrated a good correlation between self-reported medication adherence and virologic outcomes and that self-reported lower non-adherence to be more reliable than self-reported higher adherence [45,46]. Further, in the present study, adherence information was collected by the non-clinical research staff so there was less reason for participants to over-report adherence. We were also unable to relate the obtained adherence rate to CD_4_ cell count due to financial and logistical barriers to frequent laboratory monitoring, a limitation common in low and low-middle income countries.

It is also possible that selection bias occurred, as only those PLHIV who were on ART at the time of data collection were included, whereas those who were lost-to-follow up or could not attend the clinic to collect medication were excluded. The cross-sectional design of this study means we could only estimate levels of adherence at one point in time while adherence to medication is a dynamic process and participants’ behavior may change over time. In addition, the design means that casual relationships between ART non-adherence and other correlates cannot be identified and we can only demonstrate association between levels of education, illicit drug use, duration since the initiation of ART and non-adherence. Additionally, we collected PLHIV consecutively attending the care units over a limited time. Finally, the focus of this research was on individual patient factors affecting adherence. Additional research is needed to better understand heath care provider and health care system determinants of treatment adherence.

## Conclusions

The high level of non-adherence to ART found in this study (40%) is a public health concern. In view of the serious implications of non-adherence for public health, there is a critical need for targeted intervention strategies that increase the level of adherence. Non-adherence to ART in this study was associated with socio-demographic characteristics, illicit drug use, and history of taking ART. The findings of this study suggest there is a need increase the strategies used to promote adherence. It is likely that a range of interventions is needed, including increasing education, counseling, guidance on medication self-management skills and tailoring the regimen to PLHIV life styles. The feasibility of low–cost technologies such as mobile phones should also be explored. Improving adherence health care monitoring including guidelines for health care workers on monitoring adherence and strengthening peer and family support can also be effective strategies. Qualitative research is also required to understand contextual factors in identifying the optimal mix of support interventions and for who. Evaluation is also required of any interventions aimed at increasing ART to better understand cost effective measures in low-income settings.

## Competing interests

The authors declare that they have no competing interests.

## Authors’ contributions

VH designed and coordinated the study, performed analyses and wrote the manuscript. PS and VS contributed to the study design and interpretation of results and preparation of manuscript. KC, SB and FPS contributed to the data analysis and results interpretation. JD contributed to the interpretation and preparation of manuscript. All authors read and approved the final manuscript.

## Pre-publication history

The pre-publication history for this paper can be accessed here:

http://www.biomedcentral.com/1471-2458/13/617/prepub
